# Safety and efficacy of cardioneuroablation for vagal bradycardia in a single arm prospective study

**DOI:** 10.1038/s41598-024-56651-9

**Published:** 2024-03-11

**Authors:** Yafan Han, Mingliang Shao, Hang Yang, Huaxin Sun, Wanyue Sang, Lu Wang, Liang Wang, Suxia Yang, Yi Jian, Baopeng Tang, Yaodong Li

**Affiliations:** 1 Cardiac Pacing and Electrophysiology Department, The First Affiliated Hospital of Xinjiang Medical University, Urumqi, 830054 China; 2 Xinjiang Key Laboratory of Cardiac Electrophysiology and Cardiac Remodeling, The First Affiliated Hospital of Xinjiang Medical University, Urumqi, 830054 China; 3 Present Address: Medical Science and Technology Innovation Center, College of Laboratory Animals (Provincial Laboratory Animal Center), Shandong First Medical University, Affiliated Provincial Hospital, Jinan, 250117 China; 4 Present Address: Cardiovascular Department, The People’s Hospital of Xuancheng City, Anhui, 242000 China; 5 Present Address: Department of Anesthesiology, The First Affiliated Hospital of Fujian Medical University, Fuzhou, 350005 China; 6 Present Address: Department of Cardiology, The Third People’s Hospital of Chengdu, Affiliated Hospital of Southwest Jiaotong University, Chengdu Cardiovascular Disease Research Institute, Chengdu, 610014 Sichuan China

**Keywords:** Cardioneuroablation, Bradyarrhythmia, Vagus nerve, Skin sympathetic nerve activity, 3D mapping, Cardiovascular diseases, Arrhythmias

## Abstract

Cardioneuroablation (CNA) is currently considered as a promising treatment option for patients with symptomatic bradycardia caused by vagotonia. This study aims to further investigate its safety and efficacy in patients suffering from vagal bradycardia. A total of 60 patients with vagal bradycardia who underwent CNA in the First Affiliated Hospital of Xinjiang Medical University from November 2019 to June 2022. Preoperative atropine tests revealed abnormal vagal tone elevation in all patients. First, the electroanatomic structures of the left atrium was mapped out by using the Carto 3 system, according to the protocol of purely anatomy-guided and local fractionated intracardiac electrogram-guided CNA methods. The upper limit of ablation power of superior left ganglion (SLGP) and right anterior ganglion (RAGP) was not more than 45W with an ablation index of 450.Postoperative transesophageal cardiac electrophysiological examination was performed 1 to 3 months after surgery. The atropine test was conducted when appropriate. Twelve-lead electrocardiogram, Holter electrocardiogram, and skin sympathetic nerve activity were reviewed at 1, 3, 6 and 12 months after operation. Adverse events such as pacemaker implantation and other complications were also recorded to analyze the safety and efficacy of CNA in the treatment of vagus bradycardia. Sixty patients were enrolled in the study (38 males, mean age 36.67 ± 9.44, ranging from 18 to 50 years old). None of the patients had a vascular injury, thromboembolism, pericardial effusion, or other surgical complications. The mean heart rate, minimum heart rate, low frequency, low/high frequency, acceleration capacity of rate, and skin sympathetic nerve activity increased significantly after CNA. Conversely, SDNN, PNN50, rMSSD, high frequency, and deceleration capacity of rate values decreased after CNA (all *P* < 0.05). At 3 months after ablation, the average heart rate, maximum heart rate, and acceleration capacity of heart rate remained higher than those before ablation, and the deceleration capacity of heart rate remained lower than those before ablation and the above results continued to follow up for 12 months after ablation (all *P* < 0.05). There was no significant difference in other indicators compared with those before ablation (all *P* > 0.05). The remaining 81.67% (49/60) of the patients had good clinical results, with no episodes of arrhythmia during follow-up. CNA may be a safe and effective treatment for vagal-induced bradycardia, subject to confirmation by larger multicenter trials.

## Introduction

The autonomic nervous system regulates the physiological function of the cardiovascular system by maintaining a dynamic balance between the sympathetic and vagus nerves^1^. The autonomic nervous system also plays an essential role in the maintenance of sinus rhythm and blood pressure^2,3^. Excessive vagal activity may inhibit the automaticity, excitability, and conductivity of the heart, thereby negatively affecting the myocardium. Further increase of vagal activity will cause sinus node dysfunction, atrioventricular conduction disorders (such as sinus bradycardia, sinus arrest, Intermittent high atrioventricular block at night, and even vasovagal syncope^4–6^. The cardiac structure and function of these patients are generally normal, but they often have symptoms such as amaurosis and syncope^7^. Drug compliance and efficacy of the treatment of vagus bradyarrhythmia is currently poor^8^. Secondly, although a permanent pacemaker can treat vagal bradycardia and alleviate the condition, it needs frequent replacement^9^. The frequent replacement of pacemakers poses a high risk of pacemaker-related complications, such as infections, affecting the prognosis and lowering the quality of life^10–12^. Research has shown that cardioneuroablation (CNA) can block hyperactive vagus nerves by localizing and ablating the ganglionated plexus (GP)^13^. CNA is currently the primary technique for treating vasovagal syncope^14^. However, the efficacy of CNA must be largely studied in controlled clinical settings with rigorous methodology. Therefore, this study aims to evaluate the safety and efficacy of CNA in the treatment of vagus bradycardia to a new direction for exploring bradycardia caused by autonomic nervous imbalance.

## Materials and methods

### Medical ethics

The study was conducted in accordance with the Declaration of Helsinki, and all patients provided written informed consent. The study received approval from the Ethics Committee of the First Affiliated Hospital of Xinjiang Medical University (K201910-02) and was registered with the Chinese Clinical Trial Registry on 08/11/2019 under the registration number ChiCTR1900027305.

### Patient data

Sixty patients with vagus bradycardia who received CNA in the First Affiliated Hospital of Xinjiang Medical University from November 2019 to June 2022 were prospectively enrolled. All patients signed informed consent before surgery. A total of 12 lead-based electrocardiogram, Holter electrocardiogram, echocardiography, atropine test, and skin sympathetic nerve activity were performed before surgery.

### Inclusion and exclusion criteria

#### Inclusion criteria

1. Patients with clinical symptoms such as amaurosis and syncope.
2. The 12-lead electrocardiogram or Holter showed that the patient had ECG diagnosis such as sinus bradycardia and sinus arrest.
3. Patients between 18 and 50 years of age who were untreated or who did not respond to medical treatment.
4. With an abnormal increase in vagal tone confirmed by a positive atropine test.
5. Agreed to undergo CNA and regular postoperative follow-up.

#### Exclusion criteria

1. Patients with bradyarrhythmia due to hypothyroidism, myocarditis, chronic ischemic cardiomyopathy, acute myocardial infarction, etc.
2. Patients with heart failure (NYHA class III-IV) or other end-stage diseases.
3. Patients with a history of cardiac surgery, catheter ablation, or permanent pacemaker implantation.
4. Patients who refused CNA or postoperative clinical follow-up.

### Atropine test method

The atropine test is a noninvasive technique for detecting autonomic tone and is one of the preoperative evaluation criteria for CNA^15,16^. The main principle of this test is that atropine blocks acetylcholine receptors, which reduces the effect of the vagus nerve on the sinoatrial node and atrioventricular junction, promoting sympathetic innervation and increasing HR. The procedure for preoperative testing one day prior to CNA is as follows: 0.04mg/kg atropine was dissolved in 2-5ml normal saline and injected intravenously into the patients. The maximum sinus HR and heart rhythm were measured at 1, 3, 5, 7, 10, 15 and 20min after atropine injection. When the maximum HR was >90 beats/min, the atropine test was considered positive. A positive atropine test indicated that the symptomatic bradycardia may attributed to the abnormal autonomic modulation.

### CNA procedure

#### Identification of the GP

All patients received fentanyl intravenous sedation, analgesia, and anesthesia. Puncture of the left subclavian vein and the femoral vein was performed routinely. Electrophysiological data were recorded using a model 64 recorder (GE Healthcare, Waukesha, WI, USA) with a filter bandwidth of 30-500 Hz. The interatrial septum was punctured using an interatrial septum needle under X-ray fluoroscopy. Intravenous heparin anticoagulation was administered to control the thrombin time within 200-300ms. Using Carto 3 (Johnson & Johnson, New Brunswick, NJ, USA), a 4-mm ablation catheter (Johnson & Johnson, the United States) was guided to the GP by three-dimensional modeling for anatomic positioning and electrophysiological examination were performed. The targeted sites, typically known for distribution of autonomic ganglion included: the superior left ganglion (SLGP) located in the intersection area between the left atrium and/or left atrial appendage (LAA) of the left upper pulmonary vein, the right anterior ganglion (RAGP) located at the junction of the anterior wall of the right superior pulmonary vein and the left atrium. After determining the anatomic location, the distal electrode of a 4-mm head ablation catheter was applied to the intima of the left atrium for electrophysiological examination were performed (20Hz, 10-20v, 5ms) for locating the GP with the GE Healthcare Stimulation instrument (GE Healthcare, Waukesha, WI, USA). The filtering range of intracardiac electrical signals was set at 30-500Hz. The measuring screen speed was 100mm/s. Attention was paid to avoid ventricular capture and ventricular tachycardia or fibrillation during high-frequency stimulation near the mitral annulus. Transient ventricular arrest, a rapid drop in blood pressure, atrioventricular block, or at least a 10% increase from baseline in the R-R interval immediately after high-frequency stimulation was considered a positive vagal response and was labeled in the 3D model. Blood pressure, oxygen saturation, surface electrocardiogram, and endocardial bipolar potential were continuously monitored during the operation.

#### CNA process

The EP Shuttle (Johnson & Johnson, New Brunswick, NJ, USA) was guided by a 3D mapping system and thermally controlled radiofrequency ablation was performed using a 4mm ablative electrode at the pre-marked ablation site. To minimize the loss of vagal response, we ablated in the order of SLGP, RAGP. On average, 3–4 sites were ablated per GP, with the ablated area of approximately 1.2 cm^2^. The upper limit of ablation temperature was not more than 45 °C, the upper limit of power was less than 45W, and the ablation damage index was 450 for each ablation site. The criterion for successful ablation is defined as a heart rate increase of over 20% from the baseline in electrophysiological studies post-operation, and maintaining stability in repeated electrophysiological examinations 30 minutes after the surgery. For those with a CHA2DS2-VASc score of 1, a prophylactic regimen of 75mg oral aspirin was prescribed for three months to mitigate the risk of thromboembolic events.

### Noninvasive skin sympathetic nerve activity (SKNA) recording method

All patients were followed in a uniform unit and were instructed the day before recording not to smoke, drink coffee, or drink alcohol. If women were menstruating, the follow-up was postponed accordingly to minimize confounding factors affecting sympathetic activity. The recording method of Kusayama T (2020) was strictly followed^17^. The discharge signals from the skin surface of the patient's chest were recorded using Bio Amp, Power Lab amplifier, and Lab Chart Pro 8 software (AD Instruments). The filter signal range of SKNA (µV) for sympathetic discharge signal was set at 500–1000 Hz. The range of the band-pass filtering signal recorded by the ECG signal was set at 0.05–150 Hz, and the signals were captured for 30 min with the patient in a calm state. To distinguish between baseline and sympathetic discharge, the average voltage of SKNA (aSKNA) amplitude distribution ratio for each patient was plotted using JMP Pro software (SAS Institute Inc.) to show low and high-amplitude neural activity. The two neural activity groups were then fitted using the expectation-maximization method to identify two Gaussian distributions. The mean of the Gaussian curve of low-amplitude activity plus three times the standard deviation was defined as the threshold amplitude of the high-amplitude discharge activity. The neural activity above the threshold was the sympathetic discharge of each subject.

### Follow-up

**Figure 1:**
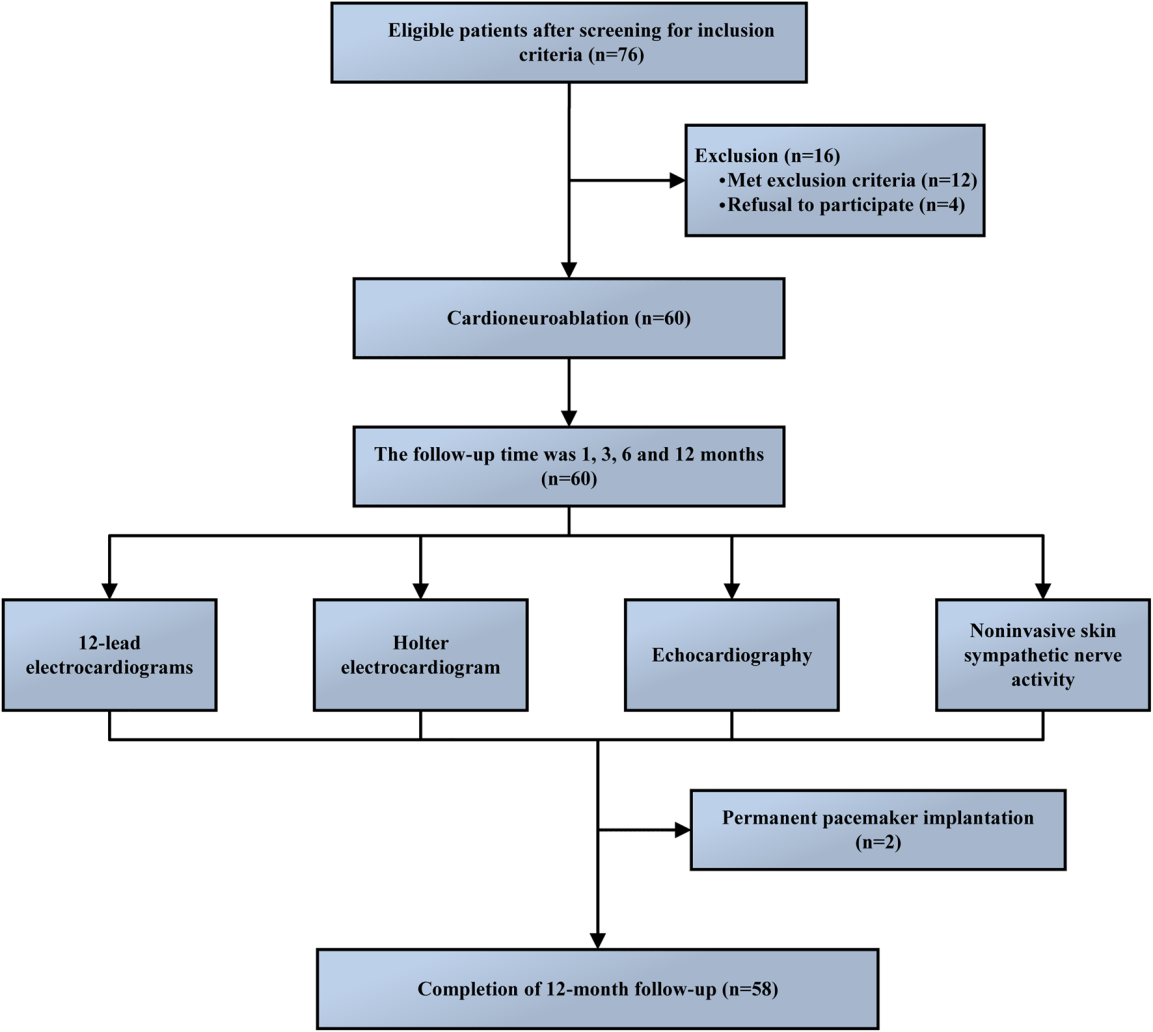
Patient Enrollment and Follow-up Process.

General data of patients, including demographic characteristics, laboratory tests, electrocardiogram, echocardiogram, and noninvasive skin sympathetic activity records, were collected before surgery. Patients were followed up in the outpatient clinic at 1, 3, 6, and 12 months post-surgery. During each follow-up visit, detailed records were made of the patients' clinical symptoms, and a series of diagnostic evaluations were conducted, including 12-lead electrocardiograms, Holter monitoring, echocardiography, and non-invasive noninvasive skin sympathetic activity assessments. The changes in HR, time domain variability, and signal changes of noninvasive SKNA were analyzed and evaluated before and after ablation. Postoperative complications, recurrence of bradycardia, pacemaker implantation, and medication were recorded and analyzed within 12 months after the operation. The primary endpoint was defined as the time to first syncope recurrence. Secondary endpoints included changes in heart rate and heart rate variability before surgery and 1, 3, 6, and 12 months after CNA surgery to comprehensively evaluate the efficacy and safety of CNA treatment. The study flow chart is shown in Fig. 1.

### Statistical analysis

The data were analyzed using SPSS 26.0 (SPSS Inc., Chicago, USA). The Shapiro-Wilk test was employed to evaluate the normality of the distribution. Data conforming to a normal distribution were reported as mean ± standard deviation (Mean±SD). Measurement treatments with non-normal distribution were expressed as median and interquartile range. Differences between groups of normally distributed variables were analyzed using the independent sample T-test. The difference in the clinical indices at different time points was analyzed using a single-factor ANOVA test. Count data (classified data) were expressed as frequency, and percentage n (%) and differences between groups of such data were analyzed using the Chi-square test. Two-tailed *P*<0.05 was considered statistically significant.

## Results

### The characteristics of the study population at baseline

This study comprised 60 patients (38 males and 22 females, mean age 36.67 ± 9.44), ranging from 18 to 50 years. The characteristics of the patients at baseline are shown in Table 1. According to the preoperative Holter electrocardiogram analysis of all enrolled patients, 28 with sinus arrest, 13 with sinus bradycardia, 9 cases were intermittent high atrioventricular block at night, 7 with both Intermittent high atrioventricular block at night and sinus arrest, and 3 with both sinus bradycardia and sinus arrest. All patients successfully underwent CNA surgery, with 7 cases experiencing postoperative sinus tachycardia (> 100 bpm), having an average age of 19.57 ± 2.44 years. These cases gradually returned to 80–90 bpm within a week after surgery. Furthermore, no postoperative complications were observed in any of the patients, including vascular injury, thromboembolism, pericardial effusion, or other surgical adverse events.

**Table 1:** Baseline data of 60 patients undergoing cardioneuroablation.

Index	Numerical value
Number of patients [n (%)]	60 (100.00%)
Male [n (%)]	38 (63.33%)
Age (years, Mean ± SD)	36.67 ± 9.44
BMI(kg/m^2^ Mean ± SD)	23.83 ± 4.47
Atropine tests were positive [n (%)]	60 (100%)
Type of bradycardia [n (%)]	
Sinus arrest	28 (46.67%)
Sinus bradycardia	13 (21.67%)
Intermittent high atrioventricular block at night	9 (15.00%)
Intermittent high atrioventricular block at night + sinus arrest	7 (11.67%)
Sinus arrest + sinus bradycardia	3 (5.00%)
Clinical symptoms [n (%)]	
Amaurosis	21 (35.00%)
Syncope	39 (65.00%)
Follow-up time (days, Mean ± SD)	368.15 ± 7.26

*BMI* body mass index, *SNRTmax* maximal sinus node recovery time, *CSNRT* corrected sinus node recovery time.

### GP localization and transesophageal cardiac electrophysiological examination

**Figure 2:**
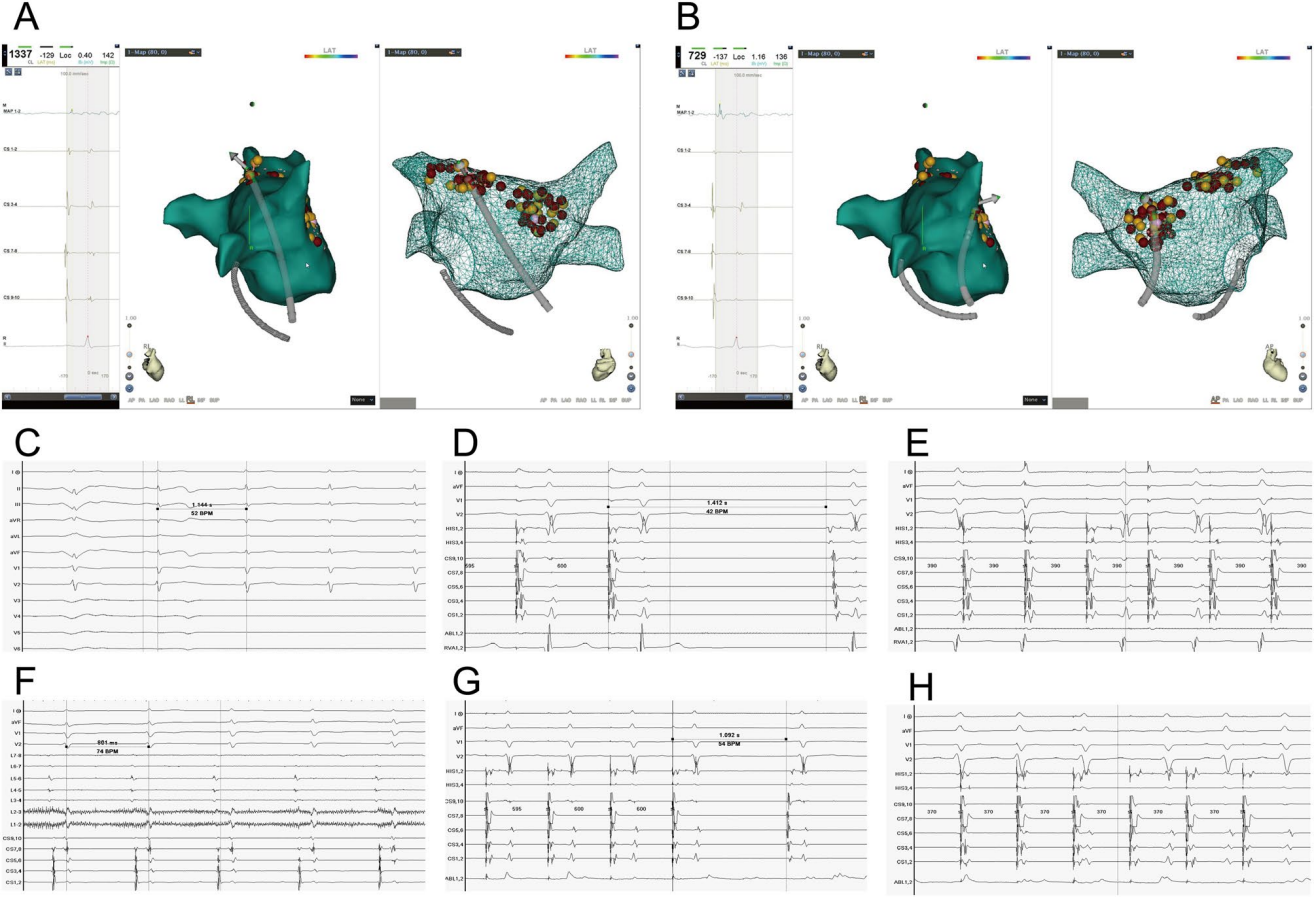
3D mapping and electrophysiological mapping during ablation. (**A**) Left superior pulmonary vein ablation, vagus reaction bradycardia; (**B**) Right superior pulmonary vein ablation, loss of vagus response and increased HR (red represents ablation targets; Yellow represents GP area; The arrow indicates the direction of catheter attachment); (**C**) Preoperative total surface electrocardiogram, basic HR 52 bpm; (**D**) The sinoatrial node function of SNRT was decreased preoperatively; (**E**) Effective Anterograde Refractory Period before cardioneuroablation; (**F**) At 20 min after the operation, the HR increased and stabilized at 74 bpm. (**G**) The sinoatrial node function of SNRT returned to normal after operation; (**H**) Effective Anterograde Refractory Period after cardioneuroablation.

The ablations are shown in Table 2, with an average of 19.3 ± 6.5 ablation sites per patient, an average ablation time of 7.9 ± 2.7 min, an average X-ray exposure of 6.2 ± 2.2 min, and a total procedure time of 48.1 ± 5.9 min. Transesophageal cardiac electrophysiological examination showed that the HR of patients was higher after CNA than before surgery (43.05 ± 7.43 vs. 78.73 ± 10.84, t = − 21.025, *P* < 0.05). The BCL, SNRT, CSNRT, and AV node WP decreased after CNA (all *P* < 0.05) (Fig. 2, Table 3).

**Table 2:** Cardioneuroablation data and follow-up of 60 patients.

Index	Numerical value
Intraoperative ablation	
Ablation discharge (times, Mean ± SD)	19.3 ± 6.5
Ablation time (min, Mean ± SD)	7.9 ± 2.7
Exposure time (min, Mean ± SD)	6.2 ± 2.2
Operation time (min, Mean ± SD)	48.1 ± 5.9
Patients with sinus tachycardia after CNA [n (%)]	7 (11.67%)
Transesophageal cardiac programming time (Month, Mean ± SD)	2.00 ± 0.80
Recurrent syncope [n (%)]	11 (18.33%)
Recurrence within 3 months [n (%)]	8 (13.33%)
The implanted permanent pacemaker [n (%)]	2 (3.33%)
Recurrent syncope time [Month, M (P25, P75)]	2 (1, 6)

**Table 3:** Esophageal electrophysiological parameters before and after cardioneuroablation.

Index	Pre-ablation	Post-ablation	Statistic	Pvalue
HR (bmp, Mean ± SD)	43.05 ± 7.43	78.73 ± 10.84	− 21.025	< 0.001
SNRT max (ms, Mean ± SD)	1852.8 ± 275.26	1293.38 ± 181.22	13.149	< 0.001
CSNRT (ms, Mean ± SD)	426.52 ± 65.58	185.18 ± 62.78	20.592	< 0.001
SACT (ms, Mean ± SD)	201.28 ± 16.46	120.52 ± 18.98	24.901	< 0.001
AV node WP (bpm, Mean ± SD)	129.38 ± 16.93	181.10 ± 19.25	− 15.626	< 0.001

*HR* heart rate, *SNRT* maximum sinus node recovery time, *CSNRT* corrected sinus node recovery time, *SACT* sinoatrial conduction time, *AV node WP* atrioventricular node Wenckebach point.

### Parameters of dynamic electrocardiogram and echocardiography

**Figure 3:**
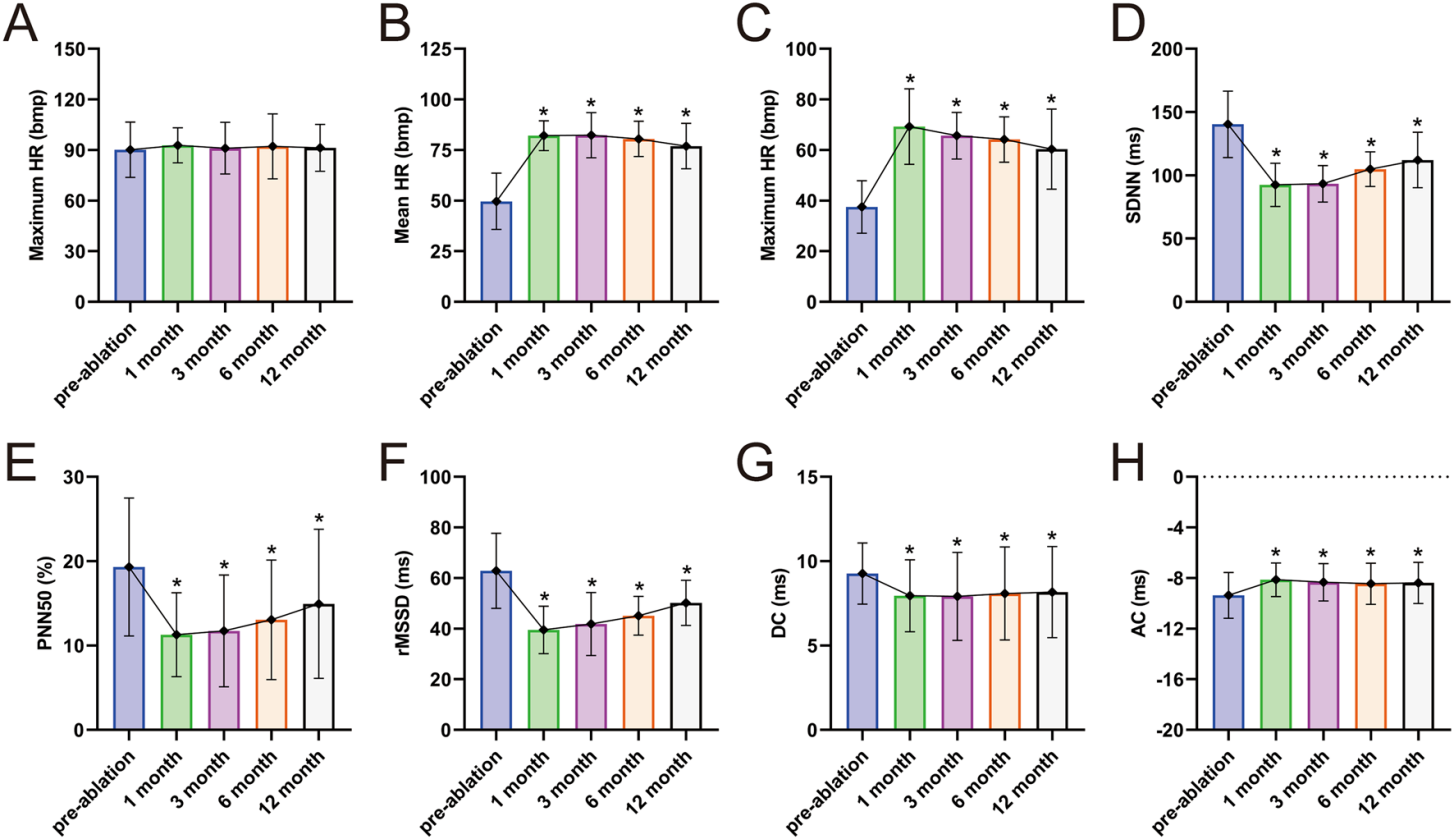
Holter electrocardiogram parameters after cardioneuroablation. (**A**–**C**) HR, heart rate; (**D**) SDNN, standard deviation of NN intervals; (**E**) PNN50, percentage of consecutive RR intervals that differ by more than 50 ms from each other; (**F**) rMSSD, root mean square of the successive differences; (**G**) DC, deceleration capacity of rate; (**H**) AC, acceleration capacity of rate. (*P < 0.05 versus pre-ablation, *n* = 58).

As shown in Fig. 3, the minimum heart rate, mean heart rate and heart rate acceleration (AC) were all higher than those before ablation, and the deceleration capacity (DC) of heart rate was lower than that before ablation within 12 months (all P < 0.05). Time domain parameters such as RR interval standard deviation (SDNN), percentage of consecutive RR interval difference greater than 50 ms (PNN50), root mean square (rMSSD) of consecutive RR interval difference were decreased 1 month after ablation compared to levels before surgery (all P < 0.05), but after 3 months of ablation, the above indexes were not statistically significant compared with those before surgery (all P > 0.05). During the follow-up period, the maximum heart rate increased slightly, and the LA, LVDD, LVEF and other ultrasound indicators fluctuated to varying degrees, but there were no statistically significant differences compared with those before operation (all P > 0.05). (Fig. 3, Table 4).

**Table 4:** Preoperative and postoperative ultrasound and skin sympathetic nerve activity parameters.

Index	Preoperative	1 month after surgery	3 months after surgery	6 months after surgery	12 months after surgery	F	P
LA (mm, Mean ± SD)	27.57 ± 4.92	25.45 ± 5.61	25.87 ± 5.30	26.73 ± 5.25	26.17 ± 5.36	1.447	0.218
LVDD (mm, Mean ± SD)	48.22 ± 4.36	47.40 ± 4.69	46.00 ± 5.60	45.52 ± 6.32	47.62 ± 3.98	2.115	0.079
LVEF (%, Mean ± SD)	67.43 ± 7.74	65.83 ± 6.85	69.58 ± 7.27	67.20 ± 7.69	66.30 ± 7.59	2.275	0.061
Burst frequency (bursts/min)	1.03 ± 0.27	2.09 ± 0.32*	1.04 ± 0.31	1.03 ± 0.29	1.02 ± 0.31	149.640	< 0.001
Burst duration (%)	10.68 ± 2.84	15.19 ± 4.12*	10.81 ± 2.90	10.04 ± 3.60	10.44 ± 3.25	23.616	< 0.001
Burst amplitude (µV)	0.87 ± 0.35	1.94 ± 0.63*	0.91 ± 0.37	0.88 ± 0.24	0.85 ± 0.19	90.081	< 0.001
Total burst area (µV*min)	0.06 ± 0.03	0.17 ± 0.10*	0.08 ± 0.04	0.06 ± 0.03	0.07 ± 0.04	45.495	< 0.001

*LA* left atrium, *LVDD* left ventricular diastolic diameter, *LVEF* left ventricular ejection fraction.

*P < 0.05 versus pre-ablation, *n* = 58.

### Parameters of noninvasive skin sympathetic nerve activity

**Figure 4:**
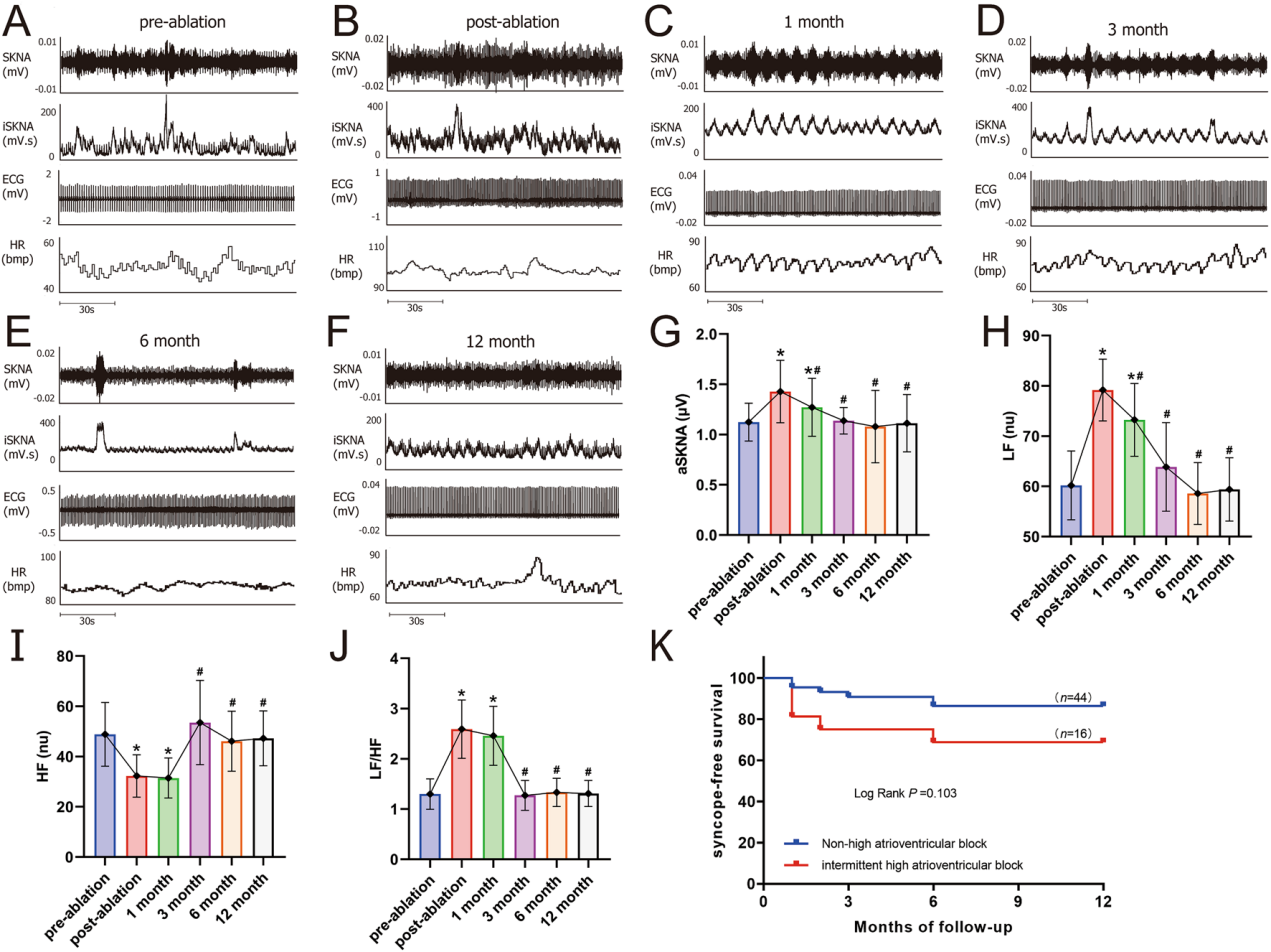
Changes in skin sympathetic nerve activity and HRV frequency domain after cardioneuroablation. (**A**–**F**) Six original images of the patient before surgery to 12 months after surgery; (**G**) aSKNA parameter comparison diagram; (**H**–**J**) Frequency domain variation of HRV. (**K**) Kaplan–Meier survival analysis curves of syncope recurrence in the two groups. *LF* Low frequency; *HF* High frequency. (*P < 0.05 versus pre-ablation; ^#^*P* < 0.05 versus post-ablation, *n* = 58).

As shown in Fig. 4 and Table 4, the aSKNA signal of sympathetic skin activity was higher after CNA than before surgery. The average number of discharges per minute after CNA, the percentage of discharge in total time, the average amplitude and the area of discharge amplitude above threshold were higher than those before operation (all *P* < 0.05). However, with the extension of follow-up time, the above indexes were not statistically significant compared with those before surgery at 3 months after surgery (all *P* > 0.05). The analysis of HRV frequency domain parameters also showed a similar trend. LF and LF/HF parameters were higher after operation, and HF parameters were lower than before operation (all *P* < 0.05). There was no statistical significance in the above indexes of heart rate variability at 3 months after surgery (all *P* > 0.05).

### Clinical outcome

During the 12-month follow-up period, compared to baseline measurements before surgery, all patients experienced significant improvements in symptoms of amaurosis and syncope. Notably, 81.67% (49 out of 60) of patients did not experience these clinical symptoms or slow heart rhythms during the follow-up period. However, 18.33%(11/60) of the patients reported at least one syncope event by the end of follow-up, and 72.73% (8/11) of the patients had recurrent syncope within 3 months of follow-up. Two patients were implanted permanent pacemakers after CNA was proved ineffective by atropine test.Furthermore, among the patients with preoperative nocturnal paroxysmal high-degree atrioventricular block, 5 cases (31.25%) experienced syncope during the follow-up period, as opposed to 6 cases (13.64%) in the group without such a history. However, this difference was not statistically significant, as demonstrated by Kaplan–Meier curve analysis (*P* = 0.103). (Table 2, Fig. 4).

## Discussion

The main findings of this study were as follows: (1) CNA rapidly alleviates clinical symptoms and effectively increases the basal HR and minimum HR; (2) CNA has the advantages of a short operation time, less trauma, high safety and no postoperative complications and adverse reactions; (3) CNA decreased the time domain and HF parameters of HRV, increased LF, LF/HF, and cutaneous sympathetic nerve activity, but returned the sympathetic nerve tone and activity to normal within three months; (4) After one year of follow-up, the majority of patients (81.67%) did not exhibit any form of bradycardia or associated clinical symptoms. These findings suggest that cardiac sympathetic denervation is a potential, safe, and effective treatment for vagal-induced bradycardia.

The 2018 ACC/AHA/HRS guidelines and 2021 ESC Guidelines recommend that pacemaker implantation should be considered in vagal bradycardia patients with syncope when they develop sinus arrest and/or high atrioventricular block after CNA and are > 40 years of age (Class I recommendation)^10,18^. However, previous studies show that patients with implanted pacemakers still experience recurrent symptoms such as vertigo or fainting, with a recurrence rate of more than 20%^19,20^. Additionally, vagal bradycardia often occurs in young and middle-aged patients who are reluctant to receive permanent pacemaker implantation^21^. There are also no oral agents that can safely and effectively reduce cardiac vagal tone for a long time. Previous studies have known that the autonomic nervous system imbalance affects the automaticity of the sinoatrial node, causing sinus bradycardia, sinus arrest, atrioventricular block, and other types of bradycardias^22,23^. Vagus bradycardia is one of the diseases that cause autonomic nervous system imbalance due to abnormal increase of vagal excitability^24,25^. Therefore, the cardiac vagus nerve has become the main target for therapeutic intervention in young patients with vagal bradycardia. CNA has the advantages of selectively damaging the autonomic nerve and eliminating the need for pacemaker implantation, which naturally becomes an important treatment for young and middle-aged patients^26^.

Recent studies increasingly support CNA as an effective treatment for symptomatic bradycardia induced by vagal activation, without significant surgery-related complications, corroborating our findings^26,27^. This suggests that CNA exerts an inhibitory effect on cardiac parasympathetic regulation, demonstrating high short-term efficacy and safety. Unlike the findings by Pachon M (2020)^26^ which showed a significant reduction in both parasympathetic and sympathetic activities two years post-CNA, our results indicated an initial increase followed by a decrease in patients' aSKNA, LF, and LF/HF ratios, with sympathetic nerve tone and activity returning to normal within three months. This discrepancy might be attributed to our ablation being limited to the SLGP and RAGP and the absence of direct validation of vagal denervation post-CNA using Extra-Cardiac Vagal Stimulation (ECVS)^28^. Beyond HRV, which is a commonly used objective measure of autonomic nerve tension, DC of heart rate also offers a non-invasive and straightforward assessment. DC is derived from the periodic changes in RR intervals of sinus rhythm, where an increased DC value indicates enhanced vagal excitability and vice versa^29,30^. Zheng L^31^ found that in patients without recurrence, DC values decreased rapidly one day post-ablation and remained below baseline levels throughout the first postoperative year, suggesting that CNA may prevent recurrence by reducing vagal nerve tension. Our study mirrored these findings, with DC values consistently lower than preoperative levels within the first year post-CNA. However, no significant difference was observed in HRV values within the same period, suggesting that DC, as a novel technique for assessing autonomic nerve tension, may offer higher sensitivity and specificity than HRV^32^. Moreover, it is noteworthy that Kaplan–Meier survival curves did not show a significant correlation between the presence of preoperative nocturnal paroxysmal high-degree atrioventricular block and an increased risk of syncope recurrence. Nonetheless, the higher incidence of syncope in patients with this condition (31.25% vs. 13.64%) cannot be overlooked. Therefore, in future studies, we will consider combining RAGP ablation with other ganglionated plexi for patients with nocturnal paroxysmal atrioventricular block^33^.

Several studies in cardiac surgery have suggested that nerve reconnection occurs after autonomic nerve fiber resection^34,35^. It takes an average of 1 to 3 years for vagal nerve reconnection, and about half a year for sympathetic nerve reconnection^36–38^. In this study, we recorded skin sympathetic nerve activity for the purpose of evaluating the overall sympathetic tone changes after GP to indirectly evaluate possible autonomic nerve regeneration. Combined with the results of this study, we suggest that clinicians should pay close attention to the sympathetic changes in patients within 3 months after CNA to prevent the recurrence of syncope and the occurrence of surgical complications. As an emerging field, CNA will see future studies integrating neurotrophic factors and other biomarkers to develop safe and effective potential techniques for neural identification, further assessing the possibility of autonomic nerve regeneration post-ablation. Concurrently, the long-term therapeutic effects of CNA necessitate extended observation and follow-up. Our future research will also incorporate long-term follow-up to investigate the enduring changes in heart rate, aSKNA, and HRV, thereby providing a more comprehensive understanding of this domain.

## Limitation

This study was conducted as a single-center, single-arm trial with a limited sample size and a relatively short follow-up duration, which may restrict the generalizability of the findings and the assessment of therapeutic outcomes. In addition, the study only ablated LSGP, RAGP, nor did it utilize ECVS techniques. These limitations could have impacted the precise evaluation of the treatment's efficacy.

## Conclusions

In this single-center study, CNA demonstrated potential efficacy in treating bradycardia induced by heightened vagal nerve excitability, suggesting it may be one of the safe and effective methods for managing such arrhythmias. However, to comprehensively assess its safety and therapeutic outcomes, further validation through clinical multicenter studies with a broader sample size is essential to ascertain the generalizability and reliability of this technique.

## Data Availability

The datasets used and/or analyzed during the current study are available from the corresponding author on reasonable request.
